# Neural Activation During Tonic Pain and Interaction Between Pain and Emotion in Bipolar Disorder: An fMRI Study

**DOI:** 10.3389/fpsyt.2018.00555

**Published:** 2018-11-06

**Authors:** Xue Han, Xiaowu Liu, Linling Li, Bo Xie, Beifang Fan, Yunhai Qiu, Tiebang Liu, Lingjiang Li

**Affiliations:** ^1^Mental Health Institute of the Second Xiangya Hospital, Central South University, Changsha, China; ^2^Department of Mental Health, Shenzhen Nanshan Center for Chronic Disease Control, Shenzhen, China; ^3^Neuroengineering Center, Shenzhen Institutes of Advanced Technology, Chinese Academy of Sciences, Shenzhen, China; ^4^Health Science Center, School of Biomedical Engineering, Shenzhen University, Shenzhen, China; ^5^Guangdong Provincial Key Laboratory of Biomedical Measurements and Ultrasound Imaging, Shenzhen, China; ^6^Research Center for Intelligent Biosensing, Research Institute of Tsinghua University in Shenzhen, Shenzhen, China; ^7^Shenzhen Key Lab for Psychological Healthcare, Shenzhen Mental Health Centre, Shenzhen, China; ^8^Clinical Psychiatry Center, National Technology Institute of Psychiatry, Changsha, China; ^9^Key Laboratory of Psychiatry and Mental Health of Hunan Province, Changsha, China

**Keywords:** bipolar disorder, pain, visual analog scale (VAS), functional magnetic resonance imaging (fMRI), insula, inferior frontal gyrus (IFG)

## Abstract

**Objective:** Pain and affective disorders have clear clinical relevance; however, very few studies have investigated the association between pain and bipolar disorder. This study investigated the brain activity of patients with bipolar disorder (BPs) undergoing tonic pain and assessed the interaction between pain and emotion.

**Methods:** Ten BPs and ten healthy controls (HCs) were exposed to emotional pictures (positive, neutral, or negative), tonic pain only (pain session), and emotional pictures along with tonic pain (combined session). A moderate tonic pain was induced by the infusion of hypertonic saline (5% NaCl) into the right masseter muscle with a computer-controlled system. Whole-brain blood oxygenation level dependent (BOLD) signals were acquired using 3T functional resonance imaging (fMRI).

**Results:** Ten BPs and ten healthy participants were included in the final analysis. During the pain session, BPs accepted more saline, but showed lower pain rating scores than HCs. When experiencing pain, BPs showed a significant decrease in the BOLD signal in the bilateral insula, left inferior frontal gyrus (IFG), and left cerebellum as compared with HCs. In the combined session, the activated regions for positive mood (pain with positive mood > baseline) in BPs were the left cerebellum, right temporal gyrus, and left occipital gyrus; the activated regions for negative mood (pain with negative mood > baseline) were the right occipital gyrus, left insula, left IFG, and bilateral precentral gyrus.

**Conclusions:** This study presents the preliminary finding of the interaction between pain and emotion in BPs. BPs exhibited lower sensitivity to pain, and the activation of insula and IFG may reflect the interaction between emotion and pain stimulus.

## Introduction

The prevalence of pain interference is elevated in patients with bipolar disorder (BPs) (1). Compared with the general population, BPs are at significantly increased risk of clinically relevant pain [Relative Risk (RR) = 2.14] (2). Furthermore, patients with affective disorders reportedly have abnormal pain perception (3). Recent studies have demonstrated that patients with major depression disorders (MDD) have decreased pain sensitivity, but both decreased and increased pain is observed in BPs. Giles et al. found BPs had decreased pain sensitivity (4); Minichino et al. found that the BD-I had reduced laser-evoked potentials (LEPs) in response to painful stimuli (5), whereas another study found that BPs had significantly lower pain tolerance (6).

Reportedly, pain and emotion are linked in many ways (7, 8); mood and emotional state alter pain perception both in healthy persons and in patients with affective disorders (9). In the experimental context, creating a positive emotional state, such as by seeing pleasant pictures or watching humorous films, generally reduces pain sensitivity. Conversely, a negative emotional state increases pain sensitivity, although these effects are less reliable than those related to positive state. Pain and affective disorders supposedly share common neuroanatomical pathways and neurobiological substrates (10). Several studies have investigated the association between pain and depression and have revealed certain common patterns of brain dysregulation in these two conditions (11, 12). Particularly, the ACC, amygdala, and PFC encode emotional and cognitive aspects of chronic pain and are also involved in processing depression. The insula is the key area for pain perception and evaluation (13), and the interaction of emotion and pain activates the insula and secondary somatosensory cortex (7).

Similar to MDD, bipolar disorders also seem associated with decreased pain sensitivity, although the evidence regarding this is insufficient. The abnormal perception may be a clinical feature of BPs. Not like the association between depression and pain has been extensively studied, only a handful of studies have examined pain sensitivity in BPs (5, 6, 14, 15), and even fewer studies have examined the interaction between pain perception and emotional state in BPs. Such studies are essential to gain a better understanding of changes occurring in the brain during pain and the interaction between emotion and pain in patients with BP. In this study, we investigated pain perception in patients with BP, and we employed functional magnetic resonance imaging (fMRI) to test brain activation.

Previous studies assessed pain after the experimental procedure considering that attention bias and testing pain after nociceptive stimulus or mood induction does not rule out the influence of covariate attention processes. This problem complicates the interpretation of neuroimaging studies reporting decreased pain ratings during cognitive task performance. An alternative task approach that limits this complication is to assess pain immediately after the distraction task, since ratings are consistent with those given in real-time (3). Hence, we used a real-time feedback experimental pain model to test pain perception and employed a well-established mood induction procedure to study the interactions between emotion and pain using fMRI. Participants first received a tonic pain stimulus or exposure to emotional stimuli only, and then they received the exact same painful stimulus during exposure to positive or negative emotional stimuli. We hypothesized that (1) compared with the healthy controls (HCs), BPs would have a lower sensitivity to pain; (2) the pain and emotional processing would have similar cellular mechanisms, the overlap areas probably include the insula.

## Materials and methods

### Participants

All the participants were recruited from the Nanshan Mental Health Center, China via advertisements. The study was approved by the local ethics committee, and all subjects provided informed consent.

All the patients were interviewed and diagnosed by experienced psychiatrists. BPs were enrolled with the following inclusion criteria: (1) bipolar I disorder consistent with the criteria of the Diagnostic and Statistical Manual of Mental Disorders, Fourth Edition (DSM-IV) based on the diagnostic assessment by the Structured Clinical Interview for DSM-IV (SCID) (16, 17); (2) age ≥ 18 and ≤ 60 years; and (3) satisfying the criteria for undergoing magnetic resonance imaging (MRI) scanning. Patients were assessed using Young Mania Rating Scale (18) and Hamilton Depression Scale (19). Exclusion criteria for HCs included: (1) any psychiatric diagnosis or organic brain disease; (2) a first-degree family history of any major psychiatric disorders, dementia, or mental retardation; and (3) a history of substance or alcohol dependence. The non-patient version of the SCID was used to ensure that HCs had no history of psychiatric or neurologic illness (20). Thus, in total, 10 healthy subjects [mean age: 31.8 (±8.0) years] and 10 patients with type I bipolar disorders [mean age: 38.6 (±9.4) years] in euthymic state were enrolled in this study.

### Clinical characterizes

Demographic, clinical, and medication regimen information are summarized in Table 1. Subjective characteristics were analyzed using SPSS software (SPSS Statistics, IBM, Armonk, NY). The groups were compared for demographic and, where applicable, clinical characteristics, including gender, age, and scale scores. Repeated Shapiro–Wilk tests were used for conducting normality tests. Clinical domains were analyzed using Chi-square test, two-sample *t*-test, or Mann–Whitney *U*-test with a confidence interval of 95%, where applicable. A *P*-value of < 0.05 was considered statistically significant.

**Table 1:** Demographic and clinical characteristics of bipolar patients and healthy controls.

	**HCs (*n* = 10)**	**BPs (*n* = 10)**	***t*/χ2**	***P***
Male/female	6/4	6/4	–	–
Age, mean (SD)	31.8 ± 8.0	38.6 ± 9.4	−1.695	0.108
Years of education, mean (SD)	16.6 ± 1.7	15.9 ± 2.0	0.843	0.411
Duration of disease, months, mean (SD)	–	14.1 ± 8.4	–	–
No. of manic episodes, mean (SD)	–	2.3 ± 1.77	–	–
No. of depressive episodes, mean (SD)	–	3.5 ± 2.17	–	–
Medications	–		–	–
SSRI + Lithium	–	7	–	–
Lamotrigine and Clonazepam	–	1	–	–
Lithium + VPA	–	2	
HAMD^*^	3.1 ± 1.0	8.9 ± 6.1	−2.817	0.002^*^
YMRS^*^	4.6 ± 1.4	5.7 ± 1.5	0.031	0.863^*^

*HC, healthy controls; BPs, patients with bipolar disorder; SSRI, Selective Serotonin Reuptake Inhibitor; VPA, Valproic Acid; HAMD, Hamilton Depression Scale; YMRS, Young Manic Rating Scale*;

* *variance uneven*.

### Experimental design

The experiment comprised three order-fixed sessions, i.e., emotional picture session (ES), tonic pain session (PS), and combined session of emotional picture and tonic *pain stimulation (CS)*.

#### ES: emotional picture session

In the ES, the experimental task consisted of presenting pictures from the standardized International Affective Pictures System (IAPS) (21, 22). Thirty pictures were selected in each category according to validated ratings (The numbers of selected pictures can be seen in Supplementary Material): positive (arousal 4.67 ± 2.34, valence 8.19 ± 1.47), negative (arousal 4.06 ± 1.25, valence 2.55 ± 1.51), and neutral (arousal 4.73 ± 1.60, valence 4.37 ± 2.51), and the pictures of the three categories were comparable for contents (human figures, daily objects, scenery, and animals). The task consisted of 6 task blocks (90 s each; two for each category of pictures) interleaved with 6 rest blocks (30 s each). Within each task block, 30 pictures of the same category were presented in a pseudo-random order and pictures were randomly presented for 2.5 s each followed by blank screen for 0.5 s. During rest blocks, participants were instructed to attend to a fixation cross. The total paradigm lasted 12 min.

#### PS: tonic pain stimulation session

PS session included a 5-min baseline, followed by a prolonged 12-min tonic pain stimulation. Tonic muscle pain was induced by infusing hypertonic saline (5%, NaCl) into the right masseter muscle using a 24-gage detaining needle with an insertion depth of ~1 cm (23). The infusion was controlled by a syringe infusion pump (Smith Medical, U.S.) installed outside the scanner room and connected to the needle via a tube. The subjects were required to rate the pain intensity every 5 s on a visual analog scale (VAS) from 0 to 10, which could be seen using the goggles they were wearing, by clicking on an MRI compatible keyboard with their left hands. The VAS rating was conducted throughout the scanning period. A standard 0.2 mL bolus of 5% hypertonic saline solution was administered over 15 s as the initial infusion for tonic muscle pain stimulus. Subsequent continuous infusion was initiated from the 120th s, and the infusion rate was adjusted every 5 s using a computer-controlled closed-loop system based on the real-time feedback of VAS scores to ensure that perceived pain intensity was maintained at an approximate preset level (VAS score: 5) to avoid habituation (24, 25). The generated muscle pain disappeared 5–10 min after the completion of infusion (26), and consecutive sessions were separated by a break lasting for at least 10 min.

#### CS: combined picture and tonic pain stimulation session

In CS, an emotional stimulation pattern similar to that in ES was followed while experiencing tonic pain stimulation using same infusion profile as that in PS.

### Behavioral data analysis

Statistical analysis of behavioral data was performed using SPSS software. The average rating of pain intensity across all rating points during tonic pain stimulation was calculated for each subject. For CS sessions, the average rating of pain intensity was calculated across all rating points during task blocks of each category of pictures (positive, negative, and neutral). Therefore, VAS scores were obtained for four conditions: pain alone; pain plus positive emotion; pain plus negative emotion; and pain plus neutral emotion. VAS scores obtained for these four conditions were compared using a two-way repeated-measures with “group” (two-levels: HCs vs. BPs) being “between-subjects” factor and “emotion” (four levels: none, positive, negative, and neutral) being “within-subject” factors. *Post-hoc* tests were performed when significant effect was noted.

### MRI data acquisition and analysis

Images were acquired using a 3.0-T MR scanner (Siemens Medical, Erlangen, Germany) at Shenzhen Institutes of Advanced Technology, Chinese Academy of Sciences. To reduce head movement, each subject's head was fixed using foam pads in a standard 12-channel birdcage head coil. First, a 3-dimensional high-resolution T_1_-weighted anatomical image (TR = 1,900 ms, TE = 2.53 ms, TI = 900 ms, FA = 9°, FOV = 240 × 240 mm^2^, matrix = 256 × 256, slice number = 176, voxel size = 0.9 × 0.9 × 1 mm^3^) was acquired. Functional T2^−^ weighted images were acquired with EPI sequence (TR = 2,000 ms, TE = 30 ms, FA = 90°, FOV = 192 × 192 mm^2^, voxel size = 3 × 3 × 4 mm^3^, slice number = 31). For each subject, three task sessions were collected.

SPM8 software (www.fil.ion.ucl.ac.uk/spm/) was used to carry out the preprocessing and statistical analysis of fMRI data. Volumes of each session were realigned to the mean volume of each session for motor correction. No subjects were excluded because of excessive head movement (>1 mm maximum displacement and 1° of angular motion). Following realignment, each subject's T1-weighted anatomical image was co-registered with the mean functional image and then segmented into gray matter, white matter, and cerebrospinal fluid using a unified segmentation algorithm. A local brain template was generated using Diffeomorphic Anatomical Registration Through Exponentiated Lie Algebra (DARTEL). Subsequently, the images were spatially normalized to the Montreal Neurological Institute (MNI) template and then resampled at a resolution of 3 × 3 × 3 mm^3^ using the normalization parameters estimated during unified segmentation, and finally smoothed with a Gaussian kernel of 6 mm full-width at half-maximum to reduce noise.

For tonic pain stimulation session (PS), preprocessed functional images were used to conduct a first-level general model (GLM) analysis to estimate the effect of tonic pain stimulation condition on brain activation of each subject separately. For task with emotional stimulation (ES and CS), first-level GLM analysis was performed to estimate the effects of the three conditions (positive, negative and neutral) on brain activation in each subject separately. To correct for head motion, the six realignment parameters were included in the design matrix as repressors of no interest. High-pass filter was applied to the data with a cut value of 128 s (for ES) and 172 s (for PS and CS) for low-frequency drifts in the signal. Finally, two contrast images were created for each subject: (1) positive > baseline, (2) negative > baseline. On the second level, one-sample *t*-test was performed to determine significant changes in signal-intensity during ES, PS, and CS sessions (*p* < 0.001, cluster-level corrected) for HCs and BPs separately. To compare the difference of signal-intensity changes between HCs and BPs, two-sample *t*-tests were performed (*p* < 0.001, uncorrected, minimum cluster size 10 voxels).

## Results

Ten BPs and ten healthy participants were included in the final analysis, the results below were obtained from small sample size.

### Saline and VAS scores in PS

**Figure 1:**
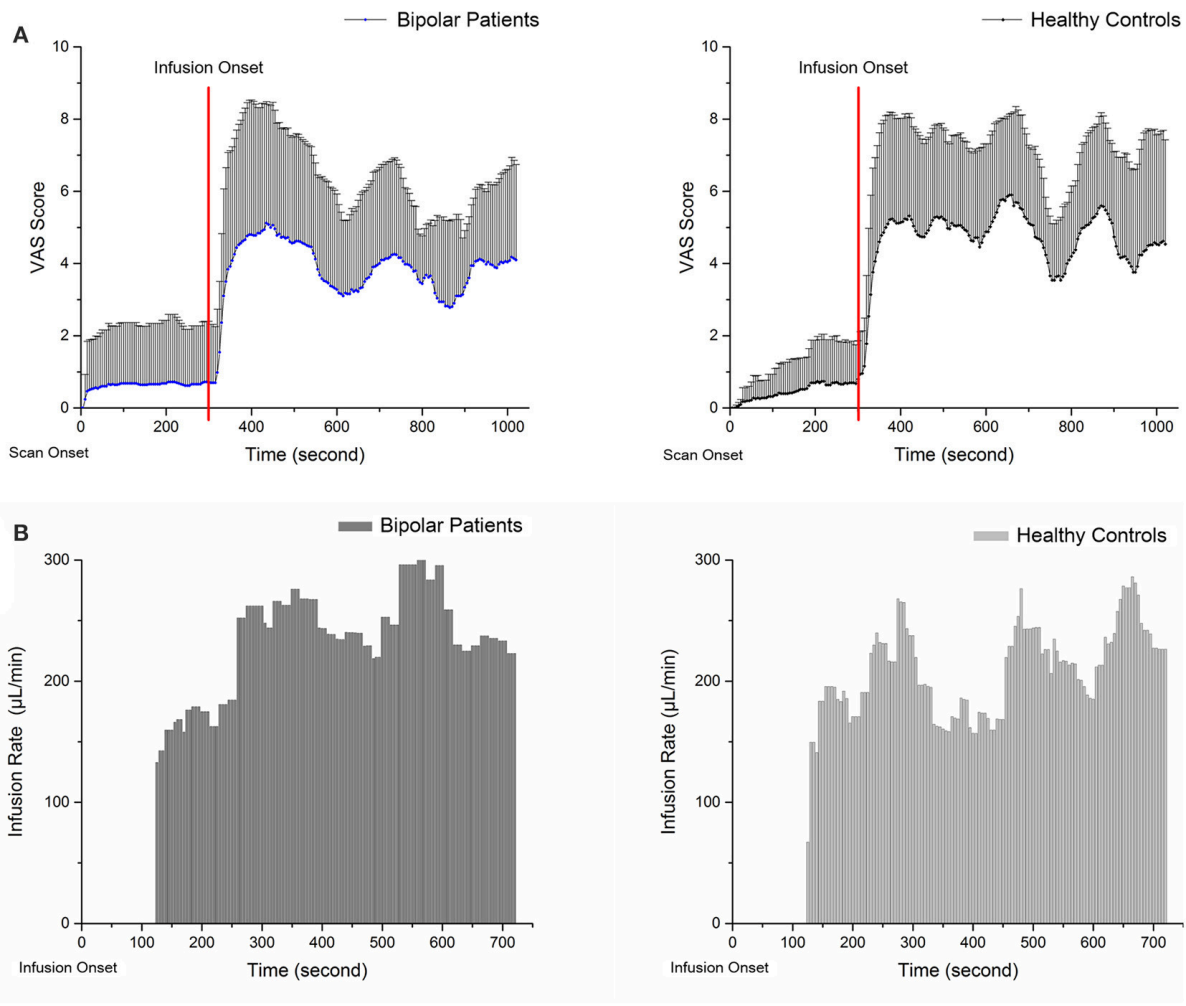
Panels **(A,B)** show the VAS scores and average infusion rate of subjects during pain session. The VAS scores were recorded from the start of the scan. **(A)** Zero to three hundred second is baseline scanning; functional scanning began at 300 s and was completely synchronized with injection. The average infusion rate was recorded from the start of injection. **(B)** At the onset of infusion, a standard 0.2 mL bolus of 5% hypertonic saline solution was administered over 15 s as a tonic muscle pain stimulus; at 120 s, infusion based on VAS score was started.

Figure 1 shows the mean pain intensity ratings and average infusion rate of the participants in PS as a function of time. VAS scores and infusion rates are described directly because the hypertonic saline infusion rate was based on the VAS score; these do not represent independent samples and cannot be compared between groups. The average pain intensity rating score in the BPs was ~3.8 (3.82 ± 0.82) vs. 4.7 (4.69 ± 0.84) in the HCs. The average infusion rate was 194.01 ± 96.6 μL/min in BPs and 172.53 ± 86.0 μL/min in HCs. The results show that BPs accepted more saline during PS, whereas their average VAS score was lower than that of HCs.

### Emotion and VAS scores

VAS scores for four conditions (pain alone; pain plus positive emotion; pain plus negative emotion; and pain plus neutral emotion) are shown in Table 2. RM-ANOVA (“group” as between-subjects and “emotion” as within-subject factors) revealed no interaction effect between “emotion” and “group.” The primary effect demonstrated that VAS scores were significantly affected by emotion (*F* = 2.92, *p* < 0.05). *Post-hoc* analysis revealed that in HCs, positive mood (*P* = 0.035) reduced pain rating scores. However, no significant difference was observed for negative mood neither in HCs nor in BPs. But, a statistically marginally significant difference was observed regarding neutral mood (*P* = 0.055) in HCs and positive mood (*P* = 0.076) in BPs.

**Table 2:** VAS scores of bipolar patients and healthy controls during various experimental sessions.

	**Pain Session**	**CS_Positive**	**CS_Neutual**	**CS_Negative**
HCs	4.67 ± 0.84	3.34 ± 0.49	3.18 ± 0.34	3.63 ± 0.99
BPs	3.82 ± 0.82	2.63 ± 0.49	3.59 ± 0.93	3.87 ± 0.68

*Data presented as mean ± standard deviation. HCs, healthy controls; BPs, patients with bipolar disorder*.

### Functional imaging results

#### ES

**Figure 2:**
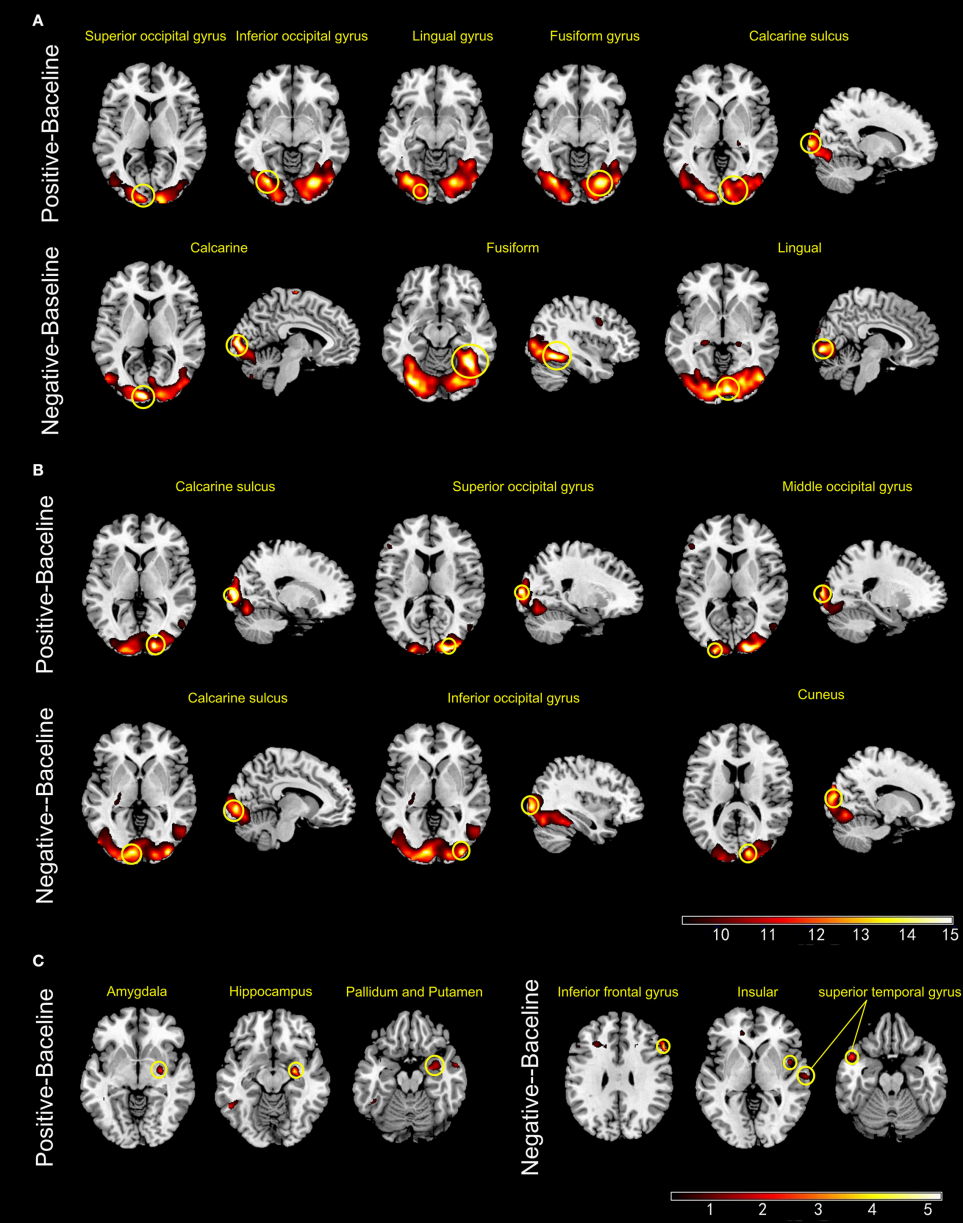
Panels **(A,B)** show the active brain regions during emotional picture sessions in healthy controls **(A)** and patients with bipolar disorder **(B)**. Panel **(C)** shows areas that exhibited significant increase in BOLD signal intensity (BPs > HCs) (*p* < 0.001, uncorrected).

During ES, the activated brain regions of the HCs for positive mood were left inferior and superior occipital cortex, left lingual gyrus, right fusiform gyrus, and right calcarine sulcus. For negative mood, the activated regions of the HCs were the left lingual gyrus, right calcarine sulcus, and right fusiform gyrus. The corresponding areas in the BPs for positive mood were the right calcarine sulcus, right superior occipital cortex, and left middle occipital cortex, whereas those for negative mood were the left calcarine sulcus, right inferior occipital gyrus, and right cuneus. The between-group contrast showed that for positive mood, BPs exhibited increased blood oxygenation level dependent (BOLD) signals in the right amygdala, right hippocampus, and basal ganglia, whereas the corresponding regions for negative mood were the right IFG, right insula, and bilateral superior temporal as compared to that in the HCs (Figure 2).

#### PS

**Figure 3:**
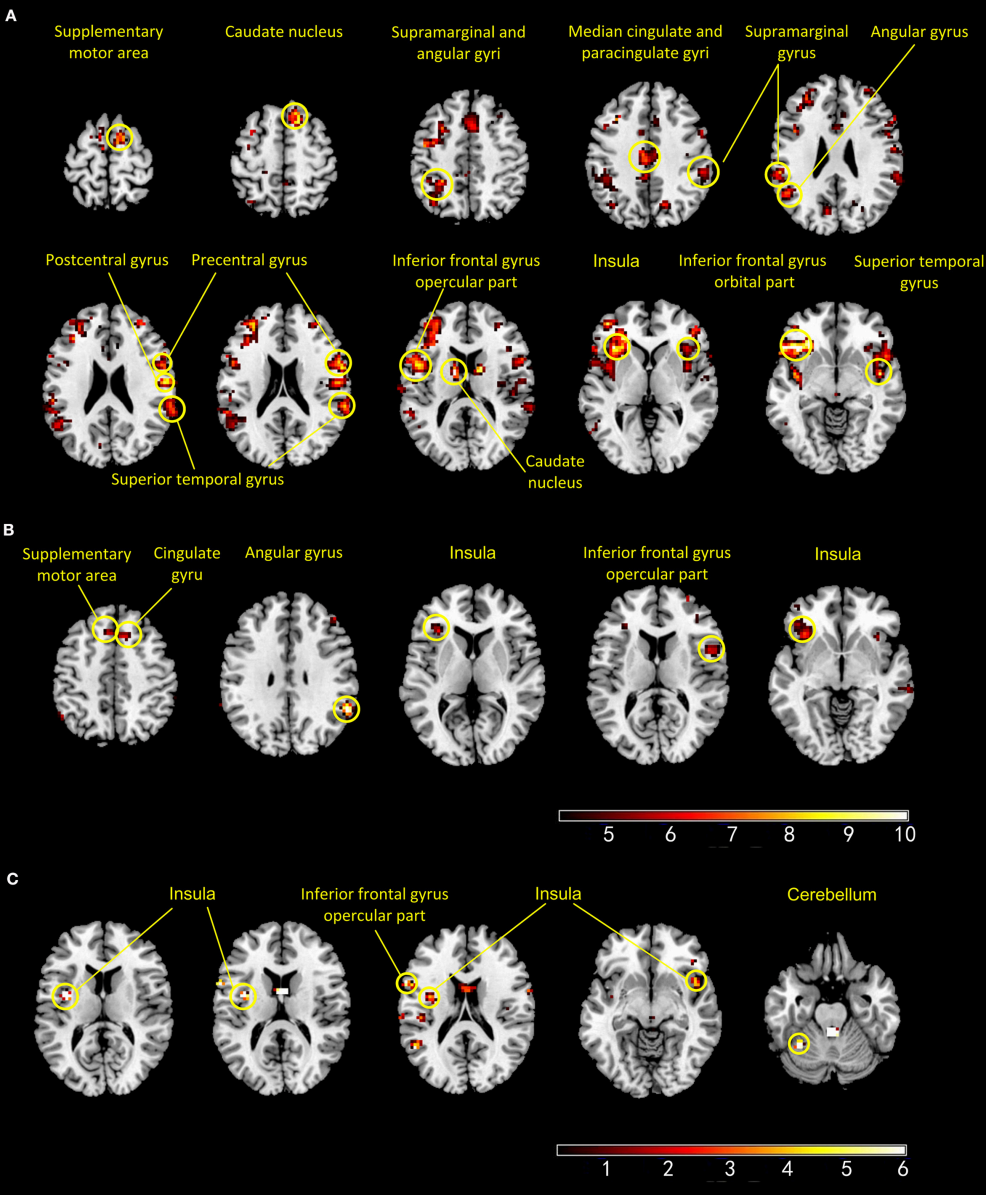
Panels **(A,B)** show the active regions during pain session in the healthy controls **(A)** and patients with bipolar disorder **(B)**. Panel **(C)** shows areas that exhibited significant increase in BOLD signal intensity (HCs > BPs) (*p* < 0 001, uncorrected).

During PS, the HCs exhibited more activated regions than those in the BPs. In the HCs, the activated brain regions included left IFG, the left insula, bilateral superior temporal cortex, right supplementary motor area, right caudate nucleus, bilateral supramarginal gyrus, left angular gyrus, and right cingulate gyrus. The brain regions activated in the BPs during PS were the right IFG, left supplementary motor area, bilateral insula, middle cingulate, and right angular gyrus. In the HCs, voxel-wise whole-brain analysis revealed significant increase in fMRI signals in bilateral insula, left IFG and left cerebellum as compared to that of the BPs (Figure 3).

#### CS

**Figure 4:**
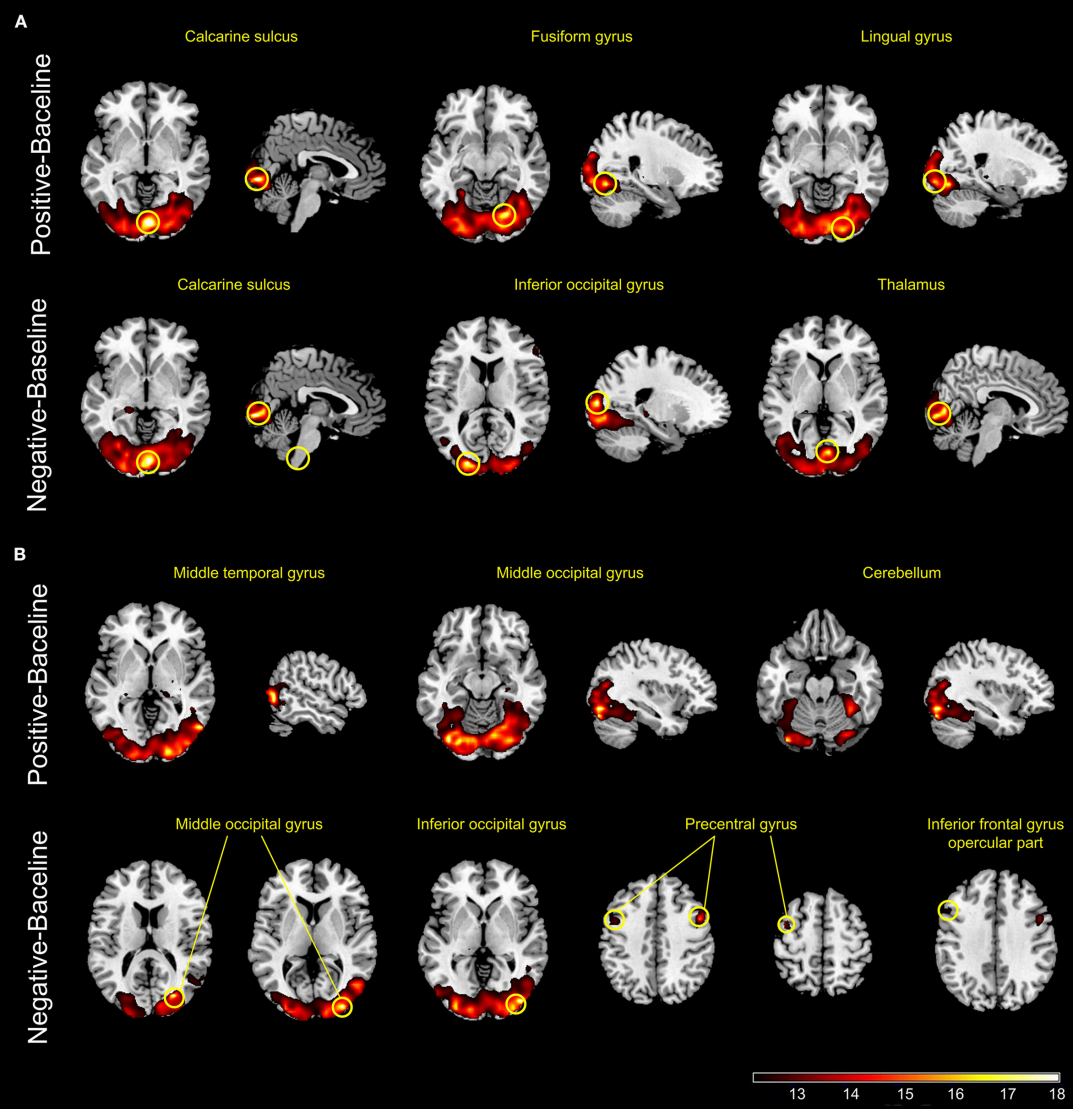
Panels **(A,B)** show the active regions during tonic pain combined with positive or negative emotional stimuli in healthy controls **(A)** and patients with bipolar disorder **(B)** (*p* < .001, uncorrected).

In the HCs, the interaction of positive emotion and pain activated right lingual gyrus, right fusiform gyrus, and right calcarine sulcus, whereas the interaction of negative emotion and pain activated right thalamus, right calcarine sulcus, and left inferior occipital gyrus. In the BPs, the interaction of positive emotion and pain activated left cerebellum, right temporal gyrus, and left occipital gyrus, whereas the interaction of negative emotion and pain activated right occipital gyrus, left insula, left IFG, and bilateral precentral gyrus (Figure 4).

Tables 3–5 summarize the significance and location of these functional imaging results.

**Table 3:** Regions of activity during emotion sessions (*p* < 0.001, uncorrected, cluster level).

	**Cluster size (No. of voxels)**	**Brain region**	**Left/Right**	**BA**	**Coordinates (MNI)**			***t*-score**
					**x**	**y**	**z**
HCs_Positive-Baseline	1035	Inferior occipital gyrus	Left	19	−30	−75	−6	11.36
		Superior occipital gyrus	Left	17	−12	−96	6	8.57
		Lingual gyrus	Left	18	−18	−87	−9	7.61
	1361	Fusiform gyrus	Right	18	24	−78	−6	10.14
		Calcarine sulcus	Right	17	15	−99	6	9.26
HCs_Negative-Baseline	3622	Lingual gyrus	Left	17	−6	−96	6	12.58
		Calcarine sulcus	Right	37	39	−57	−12	12.21
		Fusiform gyrus	Right	17	6	−87	−3	11.96
BPs_Positive-Baseline	2692	Calcarine sulcus	Right	18	18	−93	3	21.42
		Superior occipital gyrus	Right	17	21	−96	12	18.59
		Middle occipital gyrus	Left	17	−21	−99	9	16.26
BPs_Negative-Baseline	3994	Calcarine sulcus	Left	17	−9	−90	0	16.69
		Inferior occipital gyrus	Right	19	36	−90	0	16.43
		Cuneus	Right	18	15	−90	15	15.09
BPs > HCs Positive-Baseline	64	Hippocampus	Right	20	27	−9	−12	4.6
	16	Amygdala	Right	34	27	−4	−12	3.51
BPs > HCs Negative-Baseline	26	Inferior frontal cortex	Right	44	57	21	30	4.6
	13	Superior temporal gyrus	Left	21	−48	9	−21	3.56

*HCs, healthy controls; BPs, patients with bipolar disorder*.

**Table 4:** Regions of activity during pain session (*p* < 0.001, uncorrected, cluster level).

	**Cluster size (No. of voxels)**	**Brain region**	**Left/Right**	**BA**	**Coordinates (MNI)**			***t*-score**
					**x**	**y**	**z**
HCs	1436	Inferior frontal gyrus, orbital part	Left	47	−39	24	−6	9.77
		Inferior frontal gyrus, opercular part	Left	48	−45	6	12	9.54
	67	Caudate nucleus	Left	0	−12	0	15	8.75
	626	Superior temporal gyrus	Right	48	45	−3	−6	7.35
		Postcentral gyrus	Right	43	57	−12	24	7.29
	279	Caudate nucleus	Right	8	15	18	57	7.32
		Supplementary motor area	Right	6	15	3	66	6.54
	191	Superior temporal gyrus	Right	42	63	−36	21	6.78
		Superior temporal gyrus	Right	22	69	−27	21	5.85
		Supramarginal gyrus	Right	40	54	−33	39	5.63
	497	Supramarginal gyrus	Left	48	−57	−33	27	6.63
		Supramarginal and angular gyrus	Left	0	−27	−42	48	6.53
		Angular gyrus	Left	39	−48	−54	27	6.08
	101	Median cingulate and paracingulate gyrus	Right	23	−6	−18	42	6.47
BPs	39	Angular gyrus	Right	40	57	−51	30	6.14
	15	Supplementary motor area	Left	8	−2	23	47	3.94
	10	Cingulate gyrus	Right	32	5	22	41	3.46
	10	Insula	Left	47	−34	25	−4	4.23
HCs >BPs	21	Insula	Left	48	−36	−3	12	4.51
	16	Cerebellum	Left	37	−33	−51	−24	4.48
	10	Inferior frontal gyrus, opercular part	Left	44	−60	9	15	4.60
	21	Inferior frontal gyrus, opercular part	Left	44	−60	15	24	4.35

*HCs, healthy controls; BPs, patients with bipolar disorder*.

**Table 5:** Regions of activity in combined session (*p* < 0.001, uncorrected, cluster level).

	**Cluster size (No. of voxels)**	**Brain region**	**Left/Right**	**BA**	**Coordinates (MNI)**			***t*-score**
					**x**	**y**	**z**
HCs_Positive-Baseline	3839	Calcarine sulcus	Right	17	3	−84	−3	23.76
		Fusiform gyrus	Right	18	24	−72	−9	17.95
		Lingual gyrus	Right	18	21	−90	−6	14.58
HCs_Negative-Baseline	4326	Calcarine sulcus	Right	0	3	−87	−3	21.58
		Inferior occipital gyrus	Left	18	−21	−90	12	20.77
		Thalamus	Right	17	6	−78	3	17.69
BPs_Positive-Baseline	4112	Middle temporal gyrus	Right	37	54	−66	0	15.62
		Middle occipital gyrus	Left	19	−36	−78	12	15.61
		Cerebellum	Left	0	−36	−81	−21	15.2
BPs_Negative-Baseline	4448	Middle occipital gyrus	Right	19	27	−78	15	20.13
		Inferior occipital gyrus	Right	19	42	−84	0	19.17
	80	Precentral gyrus	Right	6	48	6	42	12.11
	76	Insula	Left	6	−42	0	57	6.77
		Precentral gyrus	Left	6	−45	3	45	6.33
		Inferior frontal gyrus	Left	44	−51	18	36	5.09

*HCs, healthy controls; BPs, patients with bipolar disorder*.

## Discussion

In this study, a tonic muscle pain model was used to explore pain perception and the interaction between pain and emotion. Using functional brain imaging, the neural basis of abnormal pain sensation was evaluated in the BPs.

### Effect of emotion on pain perception

Behavioral data suggests that the BPs were less sensitive than the HCs, which is consistent with that of a previous study (4). Ciaramella et al. (3) used LEPs to investigate pain perception in BPs; they also found that the BD-I showed reduced S2 activation in response to painful stimuli. Conversely, Atik et al. (6) found that the pain threshold and tolerance of the BPs was significantly lower than those of the HCs, as assessed using the cold pressor test. The differences in the results can be attributed to the use of different pain models. Unlike short-lasting pain, the physiological response to tonic pain more closely replicates chronic pain episodes experienced by patients with affective disorders (27). For brain-imaging studies, using intramuscularly injecting hypertonic saline is an established model (26) and can be more reliable as compared with the laser-pain model or cold pressor test.

A comparison of VAS scores in four conditions revealed that positive mood reduced the pain in the HCs. Similarly, positive emotional state induced by activities such as seeing pleasant pictures or watching humorous films is reported to generally reduce pain sensitivity. Most studies supported that the mood state influences the affective component of the pain rather than the sensory-discriminative aspects of pain processing. However, negative emotion did not enhance pain perception in the HCs or BPs. Besides, there is no strong evidence suggesting that positive mood may reduce pain rating scores in BPs. These results indicate that the interaction of emotion and pain in BPs is abnormal.

### Increased brain activity during mood induction

Compared with the HCs, the BPs exhibited an increased BOLD signal in the right amygdala, right basal ganglia, and right hippocampus when they viewed positive pictures and in the right IFG and right insula when they viewed negative pictures. Similar results were reported by Malhi et al. (28). Similar to the IFG, the amygdala, basal ganglia, hippocampus, and insula have been shown to be associated with emotional dysregulation in BPs (29). Recently functional studies have characterized the role of the right IFG, which appears to participate in the perception and expression of emotional information. The IFG is considered to be central to the inhibition of a prepotent response; additionally, the disruption of the right IFG has been shown to underpin response control disorders (30). Abnormal IFG activation during both emotional and cognitive tasks in BPs implied abnormal frontal-limbic activation (31).

### Decreased brain activity during PS

While experiencing tonic pain, the BPs exhibited fewer activated brain regions than the HCs. Compared with the controls, the BPs exhibited a significant decrease in fMRI signals in the insula, left IFG, and left cerebellum. These three regions are key areas for pain perception and evaluation (13). The insula are believed to be involved in consciousness and play a role in various functions linked to emotion, empathy, and pain perception. The insula receive direct pain sensory information and also transform nociceptive representation into a subjective magnitude assessment, which might modulate subsequent decisions and behavior (32). The cerebellum is classically considered to be involved in motor processing; however, it is also involved in the cognition network and pain matrix. The primary afferents transmit nociceptive (noxious) input to the cerebellum, and enhanced cerebellar activity has been demonstrated during acute and chronic pain (33). The decreased activity in the insula and cerebellum may explain lower pain sensitivity in the BPs.

Unlike the insula and cerebellum, the IFG is not associated with the pain matrix. However, the activation of the left IFG is associated with pain-related memories. In pain-free patients, the pain matrix and left IFG have been shown to be activated by the retrieval of memories related to previous painful events in the absence of any direct peripheral noxious input (34). In the BPs, the left IFG has been shown to be functionally disconnected from a network involved in emotional regulation, including the bilateral insula, ventrolateral prefrontal gyrus, superior temporal gyrus, and putamen (35). These regions are also part of the so-called pain matrix. We speculate that decreased activity in the IFG may affect the processing of pain-related information, especially affective motivation and cognitive evaluation.

### Abnormal brain activity during combined sessions

The combined sessions aimed to assess the interaction between pain and emotions. In the HCs, the interaction between positive/negative mood and pain both elicited significant activation of brain regions responsible for processing visual information and cognition, such as the occipital cortex, lingual gyrus, calcarine sulcus, and fusiform gyrus, suggesting arousal's contribution to pain modulation and evaluation. In addition, activation of the thalamus in the HCs was observed only for the interaction of negative mood and pain. The negative and positive IAPS images used in our study had relatively high arousal scores. Hence, the activation of the thalamus could be explained by the interaction of negative mood and pain. The thalamus is a key station for the transmission of nociceptive information to the cerebral cortex, and it is part of the frontal-subcortical circuits (36). We speculate that viewing negative pictures affects the perception of unpleasant feelings associated with pain; therefore, the activation of the thalamus is the result of pain processing but not the cause (11, 15, 37).

In the BPs, the interaction of positive mood and pain activated the right temporal gyrus, left occipital gyrus, and left cerebellum, which are involved in the network modulating pain processing. The interaction of negative mood and pain activated the right occipital gyrus, left insula, left IFG, and bilateral precentral gyrus. These areas contribute to both pain perception and emotional processing. The IFG and anterior insula are key areas for emotional, interoceptive, and cognitive regulation, and these areas are also associated with the perception of pain and the subsequent response, as discussed above. Considering that the BPs have lower pain sensitivity and have been underactive to the emotional stimuli in CS, these results implied a functional impairment of these areas in the BPs, especially the insula and left IFG, leading to abnormal processing of both emotion and pain. Furthermore, activated regions, such as the insula, thalamus, and cerebellum, are parts of the reward system (38). Elevated reward system responses have been reported in patients with euthymic bipolar disorder (39). Additionally, abnormal network of pain modulatory and reward systems were reported in patients with chronic pain (40). These results indicate that similar cellular mechanisms may be involved in affective disorders, pain, and reward process.

To the best of our knowledge, this is the first study to evaluate pain perception and brain activity in patients with bipolar disorder using a tonic muscle pain model. The brain changes occurring in the BPs are interesting because both decreased and increased brain activities were observed in our study. Such functional profiles could be a potential part of a cerebral signature for bipolar disorder. Our results suggest that these abnormalities may represent a novel focus of interest for research into the trait markers of bipolar disorder.

The primary limitation of our study is the small sample size. Because of the complicated and time consuming research paradigm (4.5 h for one subject on average, including interview, scales assessment, preparation of the injection and scanning), only 10 patients with bipolar disorder and same amount controls were enrolled. The relative small sample size limits the statistical power of the analysis. Moreover, a fear of needles may activate stress circuitry in the amygdala. In addition, there might be an interaction between pain perception and the drugs used by patients during the study. Previous clinical studies have suggested that antidepressants exert an analgesic effect (41). Additionally, the correlation between change in pain perception and drug and illness duration should be considered. These preliminary findings should be verified in future studies with larger sample size and stricter statistical correction threshold.

## Conclusions

In summary, this study presents the preliminary finding of the interaction between pain and emotion in BPs, both increased activation in response to emotional stimuli and decreased activation in response to painful stimuli were observed in BPs. In addition, the BPs exhibited less pain sensitivity. The abnormal pain perception in the BPs may be related to an extensive network associated with attention, sensory, executive functions, and reward processes. The activation of the insula and IFG may reflect the interaction between emotional states and an experimental pain stimulus. Our results may provide the first step in identifying brain features when BPs experience pain, and a larger sample is required in future investigations.

## Ethics statement

The IRB of Shenzhen Institutes of Advanced Technology, the Ethics Committee of Second Xiangya Hospital of Central South University and Ethics Committee of Shenzhen Nanshan Center for Chronic Disease Control approved this study. All the participants provided their informed written consent before the study.

## Author contributions

YQ, XH, and LjL contributed conception and design of the study. BX and BF assisted with the diagnosis and scales assessment. XH and XL carried out the experiments. XL and LlL performed the statistical analysis. TbL contributed to obtaining funding, language modification and experiment material. XH wrote the manuscript. All the authors contributed to the manuscript revision, read, and approved the submitted version.

### Conflict of interest statement

The authors declare that the research was conducted in the absence of any commercial or financial relationships that could be construed as a potential conflict of interest.

## Abbreviations

BPs: patients with bipolar disorder
HCs: healthy controls
BOLD: blood oxygenation level dependent
VAS: visual analog scale
fMRI: functional magnetic resonance imaging
ES: emotional picture session
PS: pain session
CS: combined session
IFG: inferior frontal gyrus.
