# Supplementary material for: Neural Activation During Tonic Pain and Interaction Between Pain and Emotion in Bipolar Disorder: An fMRI Study
Source: Front Psychiatry. 2018 Nov 6;9:555. doi: 10.3389/fpsyt.2018.00555 (PMC6232121; doi:10.3389/fpsyt.2018.00555)
Supplement: Supplementary file 1 [file Table_1.DOCX]

The numbers of the selected images from IAPS are listed below.

Positive: 1340, 1440, 1500, 1710, 1920, 1999, 2000, 2010, 2020, 2030, 2050, 2080, 2150, 2311, 2550, 5760, 5780, 5836, 7325, 8420, 8460, 8470, 8497, 8500, 8502, 8540 , 1441, 2071, 2341, 2391.

Neutral: 1112, 1670, 1675, 2102, 2190, 2210, 2214, 2221, 2272, 2396, 2397, 2493, 2516, 2579, 2580, 2595, 2830, 2840, 3210, 5535, 6900, 7002, 7006, 7038, 7175, 7211, 7150, 7506, 9070, 7010.

Negative: 2095, 2110, 2141, 2205, 2276, 2399, 2455, 2700, 2710, 2799, 2800, 2810, 2900.1, 5971, 6570.1, 8231, 9000, 9010, 904, 9140, 9180, 9181, 9280, 9472, 9520, 9530, 9560, 8010, 9001, 9561.

Positive (arousal, 4.67 ± 2.34; valence, 8.19 ± 1.47)

Neutral (arousal, 4.73 ± 1.60; valence, 4.37 ± 2.51)

Negative (arousal, 4.06 ± 1.25; valence, 2.55 ± 1.51)
